# Japanese Society of Medical Oncology/Japan Society of Clinical Oncology/Japanese Society of Pediatric Hematology/Oncology-led clinical recommendations on the diagnosis and use of immunotherapy in patients with DNA mismatch repair deficient (dMMR) tumors, third edition

**DOI:** 10.1007/s10147-023-02397-9

**Published:** 2023-08-20

**Authors:** Saori Mishima, Yoichi Naito, Kiwamu Akagi, Naomi Hayashi, Akira Hirasawa, Tomoro Hishiki, Ataru Igarashi, Masafumi Ikeda, Shigenori Kadowaki, Hiroaki Kajiyama, Motohiro Kato, Hirotsugu Kenmotsu, Yasuhiro Kodera, Keigo Komine, Takafumi Koyama, Osamu Maeda, Mitsuru Miyachi, Hiroshi Nishihara, Hiroyuki Nishiyama, Shouichi Ohga, Wataru Okamoto, Eiji Oki, Shigeru Ono, Masashi Sanada, Ikuo Sekine, Tadao Takano, Kayoko Tao, Keita Terashima, Katsuya Tsuchihara, Yasushi Yatabe, Takayuki Yoshino, Eishi Baba

**Affiliations:** 1 National Cancer Center Hospital East, Kashiwa, Japan; 2 Saitama Cancer Center, Saitama, Japan; 3 The Cancer Institute Hospital of Japanese Foundation for Cancer Research, Tokyo, Japan; 4 Okayama University, Okayama, Japan; 5 Chiba University, Chiba, Japan; 6 Yokohama City University School of Medicine, Yokohama, Japan; 7 Aichi Cancer Center, Aichi, Japan; 8 Nagoya University, Aichi, Japan; 9 The University of Tokyo, Tokyo, Japan; 10 Shizuoka Cancer Center, Shizuoka, Japan; 11 Nagoya University Hospital, Aichi, Japan; 12 Tohoku University, Miyagi, Japan; 13 National Cancer Center Hospital, Tokyo, Japan; 14 Kyoto Prefectural University of Medicine, Kyoto, Japan; 15 Keio University, Tokyo, Japan; 16 Tsukuba University, Ibaraki, Japan; 17 Kyushu University, Fukuoka, Japan; 18 Hiroshima University Hospital, Hiroshima, Japan; 19 Jichi Medical University, Tochigi, Japan; 20 National Hospital Organization Nagoya Medical Center, Aichi, Japan; 21 National Center for Child Health and Development, Tokyo, Japan

**Keywords:** Advanced solid tumor, Clinical practice guideline, Mismatch repair-deficient (dMMR), Microsatellite instability-high (MSI-H), Immunotherapy, PD-1/PD-L1 inhibitor

## Abstract

**Background:**

Clinical trials have reported the efficacy of immune checkpoint inhibitors in the treatment of mismatch repair-deficient (dMMR) advanced solid tumors. The accumulated evidence of tumor agnostic agent has been made since PD-1 inhibitor was approved and used in clinical practice. Therefore, we have revised the guideline “Japan Society of Clinical Oncology provisional clinical opinion for the diagnosis and use of immunotherapy in patients with deficient DNA mismatch repair tumors, cooperated by Japanese Society of Medical Oncology, First Edition”.

**Methods:**

Clinical questions regarding medical care were formulated for patients with dMMR advanced solid tumors. Relevant publications were searched by PubMed and Cochrane Database. Critical publications and conference reports were added manually. Systematic reviews were performed for each clinical question for the purpose of developing clinical recommendations. The committee members identified by Japan Society of Clinical Oncology (JSCO), Japanese Society of Medical Oncology (JSMO), and Japanese society of pediatric hematology/oncology (JSPHO) voted to determine the level of each recommendation considering the strength of evidence, expected risks and benefits to patients, and other related factors. Thereafter, a peer review by experts nominated from JSCO, JSMO, and JSPHO and the public comments among all societies’ members were done.

**Results:**

The current guideline describes two clinical questions and eight recommendations for whom, when, and how MMR status should be tested.

**Conclusion:**

In this guideline, the committee proposed eight recommendations for performing MMR testing properly to select patients who are likely to benefit from immunotherapy.

**Supplementary Information:**

The online version contains supplementary material available at 10.1007/s10147-023-02397-9.

## Introduction

Cancer treatment has involved a multifaceted assessment that encompasses the pathological diagnosis of the disease and an evaluation of its progression, the benefits and disadvantages of treatment, and the preferences of the patient. In diagnosing the disease, identifying the primary tumor and determining the tissue type has yielded important information that has been key to establishing a treatment plan. Recent advances in molecular biology have elucidated a variety of biological characteristics of tumors, resulting in the development and approval of tumor-agnostic drugs that transcend the organic characteristics of the disease. These guidelines have been formulated to enable the examination and treatment involved in tumor-agnostic therapy, rather than the conventional organ-specific treatment, to be smoothly implemented in the clinical setting.

The guidelines discuss the use of immune checkpoint inhibitors to treat deficient mismatch repair (dMMR) solid tumors. When new tumor-agnostic drugs are introduced clinically in the future, these guidelines will be revised in a timely manner.

## Materials and methods

The current guidelines systematically describe items to be considered when treating patients with dMMR solid tumors, including the timing and methods of testing MMR status. In the clinical setting in Japan, if appropriate tests are performed on appropriate patients and the patients receive appropriate treatment at appropriate timing based on the recommended levels described in the present guidelines, treatment outcomes in patients with solid tumors are expected to be improved.

In the preparation of these guidelines, clinical questions (CQs) were set, and regarding evidence that provides the basis for the answers to those questions, the literature was collected by handsearches and subjected to a systematic review. In setting the CQs, the working group of the Clinical Practice Guidelines for Tumor-Agnostic Genomic Medicine in Adult and Pediatric Patients with Advanced Solid Tumors (third edition) prepared draft CQs and decided which ones would be included in the guidelines.

Keywords related to each CQ were selected and sent to the Japan Medical Library Association, which generated queries used to perform comprehensive literature searches. The PubMed, Ichushi Web, and Cochrane Library databases were used in the searches. Important reports by various academic societies also were collected by handsearches and used in the guidelines. Primary and secondary screenings and systematic reviews were performed by persons in charge (SM/YN) of the working group of the Clinical Practice Guidelines for Tumor-Agnostic Genomic Medicine in Adult and Pediatric Patients with Advanced Solid Tumors (third edition). The recommendation levels specified for the CQs were determined by voting by the committee members (Table 1). The levels, which were determined based on factors such as the strength of the evidence and the expected benefits and disadvantages for patients, are as follows: strongly recommended (SR), recommended (R), expert consensus opinion (ECO), and not recommended (NR). The status of regulatory approval and insurance coverage in Japan for the treatments (including indications for testing and treatment) was not considered during the voting, but was indicated in the remarks section as needed. The overall assessments based on voting were as follows: (1) SR if ≥ 70% of the votes were for SR; (2) R if criterion (1) was not met and SR votes + R votes accounted for ≥ 70% of the total; (3) ECO if criteria (1) and (2) were not met and SR votes + R votes + ECO votes accounted for ≥ 70% of the total; and (4) NR if NR accounted for ≥ 50% of the total regardless of whether criteria (1, 2, or 3) were met. If all of the criteria (1–4) were not met, the assessment was “no recommendation level.”

**Table 1:** Degrees of recommendation and decision criteria

Degree of recommendation	Decision criteria
Strongly recommended [SR]	There is sufficient evidence and the benefits of testing outweigh the losses for patients
Recommended [R]	There is certain evidence, considering the balance between benefits and losses for patients
Expert consensus opinion [ECO]	A certain consensus has been obtained although evidence and information that shows patient benefits cannot be said to be sufficient
Not recommended [NR]	There is evidence against efficacy or for adverse outcome, generally not recommended

The recommendations for the CQs include recommendations that are not currently based on strong evidence. As new evidence accumulates, the information and recommendations in these guidelines may change significantly. Although these guidelines will be updated as appropriate, in using a drug clinically, the latest medical information should be reviewed, and every effort made to ensure the drug is used properly.

## Results

### Cancer and mismatch repair function

Repairing non-complementary base pairs (mismatch) that are produced during DNA replication (mismatch repair: MMR) is an essential function for maintaining genome homeostasis. The condition where the MMR function is reduced is described as MMR deficient (dMMR), and the condition where the MMR function is maintained is described as MMR proficient (pMMR). The reduced MMR function changes the number of repeats of one-base to several-base repeat sequences (microsatellites). This phenomenon is called microsatellite instability (MSI). Decreased MMR function increases the likelihood of changes in repetitive sequence regions present in coding regions of genes involved in cancerous changes, including tumor suppression, cell proliferation, DNA repair, and apoptosis. An accumulation of these genomic alterations is thought to play a role in tumorigenesis and tumor growth [1]. The condition where microsatellite instability is detected with a high frequency is described as MSI-high (MSI-H), and the condition where microsatellite instability is detected with a low frequency or not detected is described as MSI-low/microsatellite-stable (MSI-L/MSS).

The causes of cancers in which decreased MMR function is seen generally vary according to the type of cancer. A common cause of sporadic dMMR tumors is acquired hypermethylation [2], mainly in the MLH1 promoter region [1]. Other known causes include MMR gene sequence changes and decreased expression as a result of abnormal methylation of a promoter region [1]. When pathological variants of MLH1, MSH2, MSH6, and PMS2 or deletion of EPCAM [3–5], which is adjacent to the upstream region of MSH2, are seen in 1 allele in the germline, this is referred to as Lynch syndrome. Tumors that occur as a result of these genomic alterations are referred to as Lynch syndrome-related tumors (see Sect. 6. Lynch syndrome [6, 7]). Constitutional mismatch repair deficiency (CMMRD) syndrome, where a pathogenic germline variant is seen in both alleles of an MMR gene, also has been reported as a rare disorder and is known to be associated with diseases such as colorectal and small intestine cancer, acute leukemia, and brain tumors (medulloblastomas and high-grade gliomas) beginning in childhood [8]. The condition in which the incidence of comorbidities other than gastrointestinal cancers, especially brain tumors, is high is known as Turcot syndrome, which is characterized by the occurrence of medulloblastomas and high-grade gliomas.

### Frequencies of dMMR solid tumors by cancer type

dMMR solid tumor is seen in a variety of organs, and its incidence varies greatly according to race and ethnicity, cancer type, disease stage, and whether it is hereditary or sporadic. Depending on the report, there is significant variability in the incidence of dMMR solid tumors detected by MSI or ICH testing (for the testing method, see Section “4. dMMR testing methods”), depending on aspects such as the population tested and the test method. The results of an analysis of MSI tests of 26,469 patients with unresectable or recurrent solid tumor in Japan performed between December 2018 and November 2019 showed an overall incidence of high-level MSI (MSI-H) of 3.72%. In cancer types for which analyses were performed for at least 100 patients, the incidence of MSI-H, in the descending order, was 16.85% in endometrial cancer, 8.63% in small intestine cancer, 6.74% in stomach cancer, 5.60% in duodenal cancer, and 3.78% in colorectal cancer [9].

There have been several studies of the tumor-agnostic incidence of dMMR solid tumors that have used NGS (for the testing method, see Section “4. dMMR testing methods”). In a total of 11 cancer types with a high incidence in 12,019 patients with 32 types of solid tumors, MSI-H was seen in approximately 10% of patients with Stage I–III disease and approximately 5% of patients with Stage IV disease [10]. In addition, Memorial Sloan Kettering Cancer Center (MSKCC) performed NGS of tumor and normal DNA using the MSK-IMPACT platform and evaluated dMMR using MSIsensor, a computer analysis algorithm that compares tumor and normal DNA pairs and reports the percentage of unstable microsatellite regions detected as a cumulative score. With this algorithm, MSI-H was defined as an MSIsensor score ≥ 10 points, indeterminate (MSI-I) as ≥ 3 and < 10 points, and MSS as a < 3 points. An analysis that included more than 50 types of solid tumors in 15,045 patients found the incidence of MSI-H, MSI-I, and Lynch syndrome-related tumors, which are shown in Table 2 [11].

**Table 2:** Prevalence of Lynch syndrome by cancer type and MSI status

Cancer type	Total	MSI-H/I* (%)	%MSI-H/I Lynch
Total count	15,045	326 (2.2)	53 (16.3, 0.35)
Colorectal	826	137 (16.5)	26 (19.0, 3.1)
Endometrial	525	119 (22.7)	7 (5.9, 1.3)
Small bowel	57	17 (29.8)	2 (11.8, 3.5)
Gastric	211	13 (6.1)	2 (15.4, 0.9)
Esophageal	205	16 (7.8)	0 (0, 0)
Bladder/urothelial	551	32 (5.8)	12 (37.5, 2.2)
Adrenal	44	19 (43.1)	2 (10.5, 4.5)
Prostate	1048	54 (5.1)	3 (5.6, 0.29)
Germ cell	368	33 (9.0)	1 (3.0, 0.27)
Soft tissue sarcoma	785	45 (5.7)	2 (44.4, 0.25)
Pancreatic	824	34 (4.1)	5 (14.7, 0.61)
Mesothelioma	165	6 (3.6)	1 (16.7, 0.61)
CNS tumors	923	30 (3.3)	1 (3.3, 0.11)
Ovarian	343	46 (13.4)	0 (0, 0)
Lung	1952	94 (4.8)	0 (0, 0)
Renal cell	458	11 (2.4)	0 (0, 0)
Breast	2371	150 (6.3)	0 (0, 0)
Melanoma	573	25 (4.3)	1 (4.0, 0.17)
Other cancer type**	2816	144 (5.1)	0 (0, 0)

*MSI-I MSI-Indeterminate

**Other cancer type includes less common tumors, the majority of which were ampullary carcinoma, anal carcinoma, appendiceal carcinoma, osteosarcoma, peripheral nerve sheath tumor, choriocarcinoma, cervical cancer, neuroendocrine tumor, neuroblastoma, thymic tumor, pheochromocytoma, vaginal carcinoma, Wilms tumor, cancer of unknown primary, head and neck cancer, hepatocellular carcinoma, cholangiocarcinoma, chondrosarcoma, Ewing sarcoma, non-Hodgkin lymphoma, leukemia, and retinoblastoma

### Clinical picture of dMMR solid tumors

The association between the conditions of microsatellites and prognoses was weak in a study of 18 types of dMMR solid tumors (5930 cancer exomes) [12]. Besides this study, the outcomes of dMMR solid tumors in various cancers have been analyzed. However, the association with prognoses has not been elucidated.

The clinical picture of dMMR solid tumors will be described by the type of cancer below.

#### Clinical picture of dMMR gastrointestinal cancer

In Europe and the USA, 13% of all colorectal cancers have dMMR [13], and in Japan, 6–7% have dMMR [14, 15]. Among stage IV cancers, however, the incidence is low and is reported to be 1.9–3.7% in Japan [16, 17]. Approximately 20–30% of dMMR colorectal cancers are related to Lynch syndrome, and approximately 70–80% are sporadic. Both types of cancers occur commonly in the right-side colon and have a high percentage of poorly differentiated adenocarcinomas.

As for the association with prognoses, it has been reported that the prognoses of stage II patients are good and the prognoses of patients for whom curative resection is not possible are poor. The *BRAF* V600E mutation is detected in 35–43% of dMMR colorectal cancers [18], but is rare in Lynch syndrome-related colorectal cancers even though they have dMMR [2], as shown in Table 3.

**Table 3:** Clinical picture of dMMR solid tumors

	Clinical picture
dMMR colorectal cancer	
Lynch-associated	More common in juvenile・multiple cancer (synchronous and metachronous)・right-sided colon・poorly differentiated adenocarcinoma
Sporadic	More common in elderly female right-sided colon poorly differentiated adenocarcinoma
dMMR hepato-biliary-pancreatic cancer	
Lynch-associated	Bile duct cancer: good prognosis Pancreatic cancer: good prognosis
Sporadic	Hepatocellular carcinomas: high-grade malignancy Bile duct cancer: more common in juvenile Pancreatic cancer: good prognosis
dMMR gynecological cancer	
Lynch-associated	The second among the most frequent in women and more common in juvenile Endometrial carcinomas known to occur in the uterine isthmus are common (clear cell carcinoma/serous carcinoma/carcinosarcoma may also occur) Carriers of the MSH6 pathogenic variant are recognized as having a comparatively high risk of endometrial cancer
Sporadic	Low grade (well-differentiated) endometrioid carcinoma is more common [42, 43]
dMMR urological cancer	
Lynch-associated	Urothelial cancer: more common in juvenile. The risk of developing the disease in women increases to the same level as in men Prostate cancer and germ cell carcinoma are also lynch-associated cancer
Sporadic	Unknown

The incidence of dMMR tumors in all gastric cancers is high, being approximately 20–25% in Europe and the USA and approximately 8–19% in Asian countries [19]. It has been reported that dMMR gastric cancer commonly occurs in elderly women; its main type is distal, intestinal-type adenocarcinoma, and lymph node metastasis and *TP53* mutations are rarely seen [20]. It has also been reported that the prognosis of MSI-H gastric cancer is better than that of MSI-L/MSS gastric cancer (HR 0.76) [21].

The incidence of dMMR solid tumors in all small intestine cancers is relatively high, being 5–45% [22]. There are only a few reports about esophageal cancer, and no specific views on the incidence or prognosis have been established.

#### Clinical picture of dMMR hepato-biliary-pancreatic cancer

Among hepato-biliary-pancreatic cancers, the incidence of dMMR tumors is low and there are a limited number of comprehensive reports. In hepatocellular carcinomas, 1–3% are dMMR tumors, which are found not only in advanced cancers but also in early cancers [4]. It has also been reported that they are high grade and recur in a short period of time [23]. In biliary tract cancers, the incidence of sporadic MSI-H tumors is reported to be 1.3% [25]. They often develop at a young age [24] and are found among both early and advanced cancers [25]. One report showed that MSI-H tumors had better prognosis than MSS tumors [26], while another report showed that there was no difference in prognosis between these two types of tumors [25]. Thus, there are no consistent views.

Although it was reported from Japan that the incidence of dMMR in pancreatic cancers was 13% [27], recent reports from overseas showed the incidence is 0.8–1.3% [28–31]. Therefore, it is assumed to be around 1% currently. There are some reports showing good prognoses [29, 30], and it is said that dMMR tumors readily respond to immune checkpoint inhibitors [30]. There is also a report that the time to recurrence did not differ between patients receiving and not receiving an adjuvant therapy [32], and another report showed that dMMR pancreatic cancers were poorly differentiated and wild-type *KRAS* was frequently expressed in them [27]. However, the clinical significance of these findings has not yet been elucidated. Clinical picture of dMMR hepato-biliary-pancreatic cancers is summarized in Table 3.

#### Clinical picture of dMMR gynecological cancer

In gynecological cancers, dMMR is most commonly seen in endometrial cancer. In the general population, the lifetime risk for endometrial cancer is 3%, while in patients with Lynch syndrome, it is 27–71% [33]. In endometrial cancers, the incidence of dMMR is 20–30%. Approximately 5–20% of these patients have Lynch syndrome (with pathogenic variants of the MMR gene in the germline), while approximately 80–90% of them are sporadic [34, 35]. A comparison of the clinical features of Lynch syndrome-related gynecological cancers and sporadic gynecological cancers is summarized in Table 3. The analysis of 173 patients with endometrial cancers reported that progression-free survival (PFS) and overall survival (OS) in patients with dMMR endometrial cancers tended to be poorer than those in patients with proficient MMR (pMMR) endometrial cancers (PFS: *P* = 0.057; OS: *P* = 0.076), while in patients with Lynch syndrome, there was no association with prognoses (PFS: *P* = 0.357; OS: *P* = 0.141) [36].

Regarding ovarian cancer, although the lifetime risk is 1.5% in the general population, it is 3–20% in patients with Lynch syndrome [33, 37, 38]. In Japan, pathogenic variants of MMR genes are seen in approximately 2.6% of patients with epithelial ovarian cancer [39].

The risk of developing Lynch syndrome-related tumors varies depending on the gene, and the risk of developing endometrial cancer is known to be relatively high in patients with *MSH6* pathogenic variants [40, 41].

#### Clinical picture of dMMR urological cancer

Of urological cancers, dMMR is most commonly seen in renal pelvic/ureteral cancers and also seen in prostate cancer, germ cell tumor, and bladder cancer. In renal pelvic/ureteral cancers, the frequency of dMMR is 5–11.3% [44]. Deficient DNA mismatch repair renal pelvic/ureteral cancers are histopathologically characterized by an inverted growth pattern and a low stage, while there are no sites of predilection for these cancers [45]. Lynch syndrome-associated renal pelvic/ureteral cancers develop at a younger age as compared with general pelvic/ureteral cancers, and the risk of developing it increases in women to a level equal to that in men [46]. There is also a report that more than half of Lynch syndrome-related renal pelvic/ureteral cancers are MSS/MSI-L [46]. Besides renal pelvic/ureteral cancers, it has been reported that some prostate cancers, germ cell tumors, and bladder cancers may be related to Lynch syndrome [44]. Clinical features of sporadic dMMR urological cancers are not known. Clinical picture of dMMR urological cancer is summarized in Table 3.

### dMMR testing methods

The dMMR testing methods include MSI testing, the immunohistochemistry (IHC) for MMR proteins (MLH1, MSH2 MSH6, and PMS2), and NGS testing, as shown in the following.

#### MSI testing

In the MSI testing method, microsatellite regions of DNA obtained from normal and tumor tissues are amplified by the PCR method and the number of repeats of microsatellite sequence is determined and compared. In practice, the length of PCR products, which reflect the number of repeats, is compared in electrophoresis. In a method using a classical Bethesda panel, the length of five microsatellite markers (BAT25, BAT26, D5S346, D2S123, and D17S250) is compared between tumor and normal tissues. When PCR products with different lengths are detected, MSI is determined to be positive, positive MSI for two or more markers is determined to be MSI-H, and positive MSI for only 1 marker is determined to be MSI-L (low-frequency MSI). When no positive MSI is observed for any marker, it is determined to be MSS (microsatellite stable). MMR function in a tumor is judged to be deficient (dMMR) for MSI-H tumors and as proficient (pMMR) for MSI-L/MSS tumors. The Bethesda panel contains three dinucleotide repeat markers, which have been reported to be less sensitive to MSI than mononucleotide repeat markers. In recent years, in dMMR testing, panels consisting of only mononucleotide repeat markers [pentaplex and the MSI test kit (FALCO)] are often used. BAT25 and BAT26, mononucleotide repeat markers used in many panels, are high in both sensitivity and specificity for MSI [47].

**Fig. 1:**
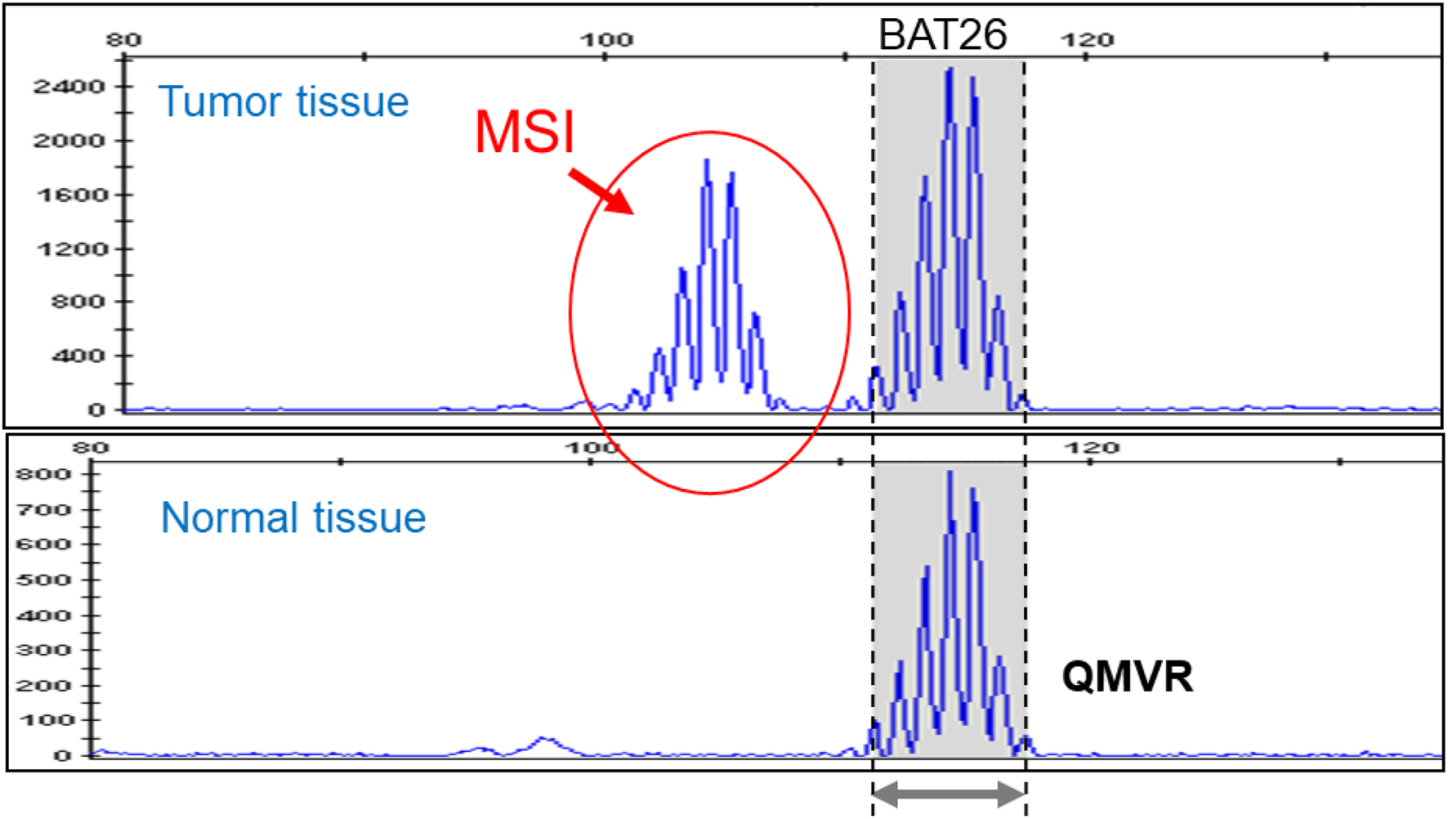
MSI analysis of BAT26. Area with a gray background was QMVR of BAT26. In tumor tissue, the sizes of microsatellites (patterns framed by red lines) are different from those seen in normal tissue

In September 2018, “MSI test kit (FALCO)” received regulatory approval in Japan. As of June 2021, it had been approved for the following indications: “assistance in evaluating whether pembrolizumab is indicated in patients with solid tumors,” “assistance in evaluating whether nivolumab is indicated in patients with cancer of the colon or rectum,” “assistance in diagnosing Lynch syndrome in colorectal cancer,” and “assistance in selecting chemotherapy for colorectal cancer.” This test kit adopts a panel consisting of only mononucleotide repeat markers (BAT-25, BAT-26, NR-21, NR-24, and MONO27) (Table 4). These markers display quasi-monomorphism, and the quasi-monomorphic variation range (QMVR) of each marker is within constant limits irrespective of race (Table 5) [48]. The lengths of the PCR products of the microsatellite markers for normal tissue fall in the range of a mean of ± 3 bases (QMVR) in the MSI test kit (FALCO). Therefore, by defining a marker with a length outlying the QMVR as being MSI-positive (Fig. 1), MSI status can be evaluated using only tumor tissues. Actually, for many solid tumors, the MSI-H status determined only with a tumor tissue was consistent with that determined with a pair of normal and tumor tissues [49].

**Table 4:** Panel for MSI testing

MSI testing (FALCO)	
Marker sequencing structures	
BAT25	Mononucleotide repeats
BAT26	Mononucleotide repeats
NR21	Mononucleotide repeats
NR24	Mononucleotide repeats
MONO27	Mononucleotide repeats

**Table 5:** Quasi-monomorphic variation range (QMVR) decided by 149 specimens from healthy Japanese individuals

	NR21	BAT26	BAT25	NR24	MONO27
Japanese	98.4–104.4	111.4–117.4	121.0–127.0	129.5–135.5	149.9–155.9
Patil DT et al. [48]	98–104	112–118	121–127	129–135	149–155

**Fig. 2:**
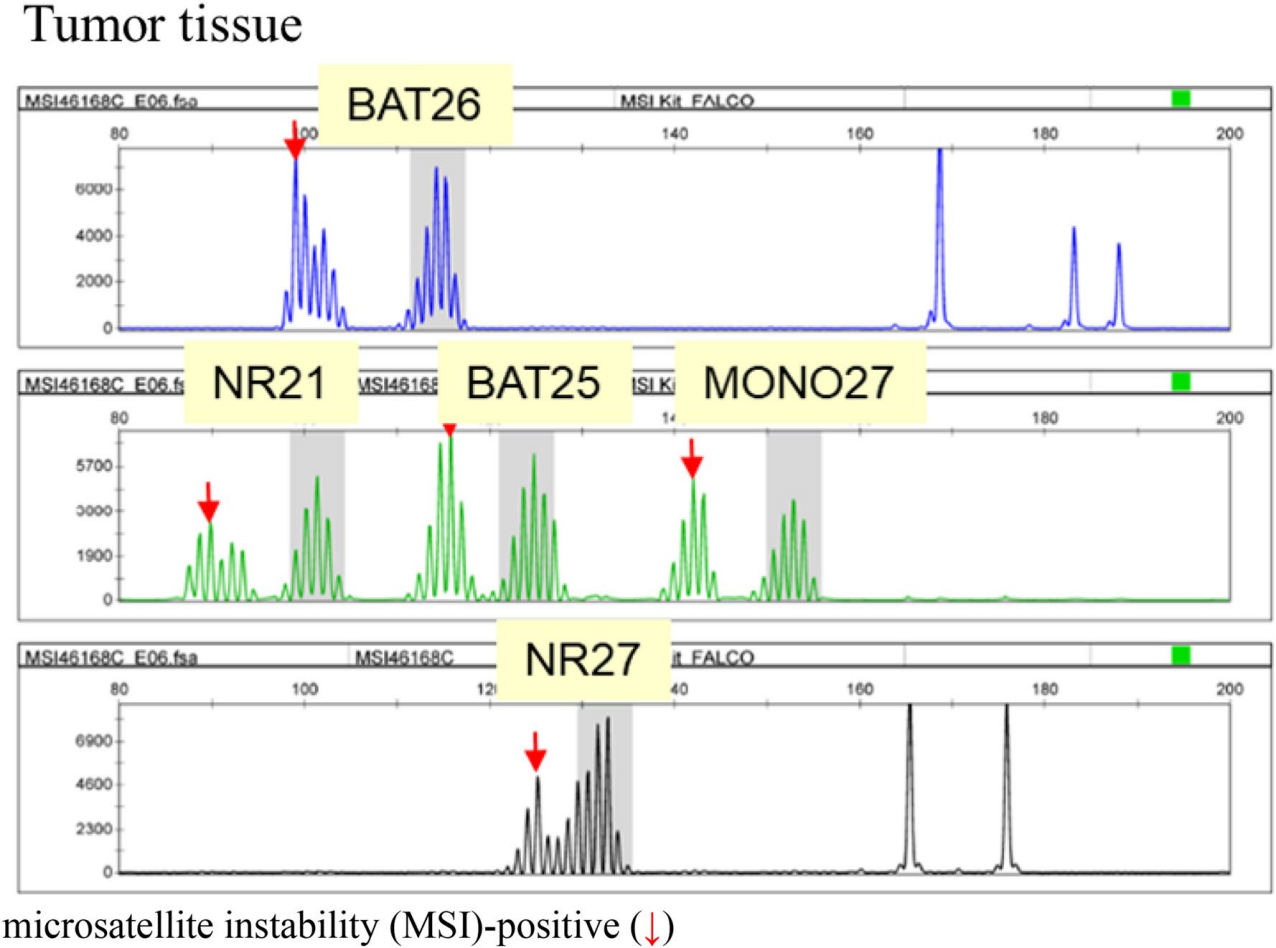
MSI-H case (colorectal cancer). Microsatellite instability (MSI)-positive (down arrow)

**Fig. 3:**
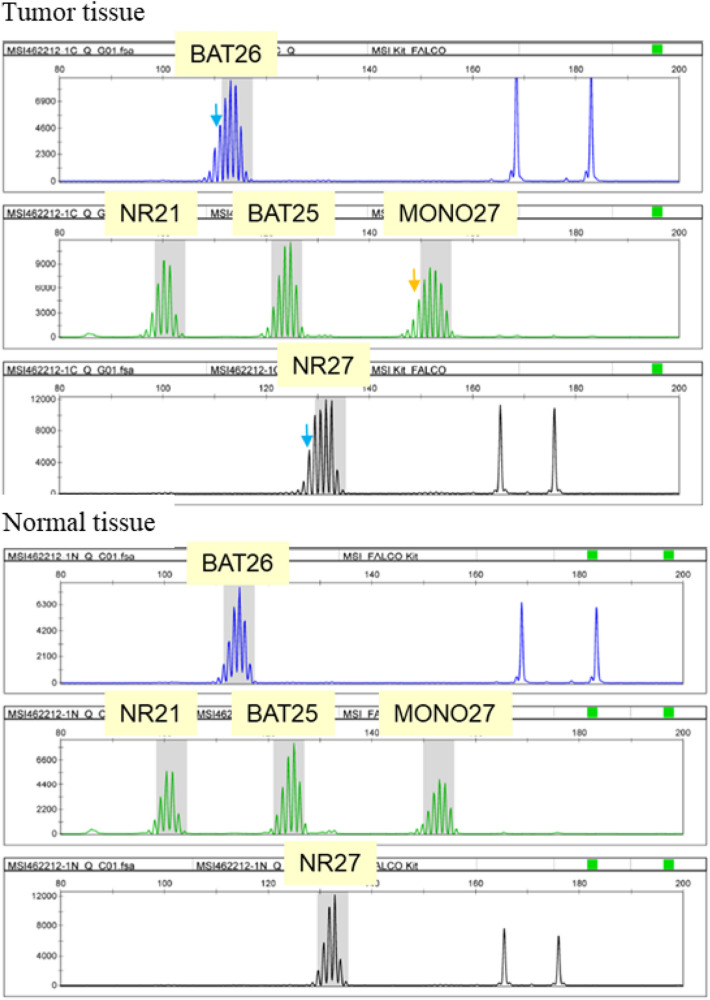
MSI-H case that need attention in decision (endometrial cancer). In tumor tissue, there were two marker (down arrow) that need attention in decision. In comparison with markers in normal tissue, these patterns were defined as MSI-positive. Moreover, there was one additional marker defined as MSI-positive compared with normal tissue

For colorectal cancer, the concordance rate of the dMMR determination between MSI testing and the IHC for MMR proteins [refer to “4.2 Immunohistochemistry (IHC) testing for MMR proteins”] has been reported to be ≥ 90%. However, some solid cancers other than colorectal cancer have shown slightly low concordance rates. As a possible cause for this finding, it has been suggested that the extent of altered repeat sequences may vary among organs: On average, a 6-base shift is observed for colorectal cancer (Fig. 2), while only a 3-base shift is observed for other solid tumors (Fig. 3) [50]. The MSI test kit (FALCO) uses the QMVR of the mean ± 3 bases as a criterion for evaluating each marker. Therefore, if the extent of the shift is small, MSI will test false-negative. Such false negative results have been reported for brain tumor, ureteral cancer, endometrial cancer, ovarian cancer, bile duct cancer, and breast cancer. Therefore, when an MSI test is performed with tumor tissue alone, the results must be interpreted carefully.

#### Immunohistochemistry (IHC) testing for MMR proteins

**Fig. 4:**
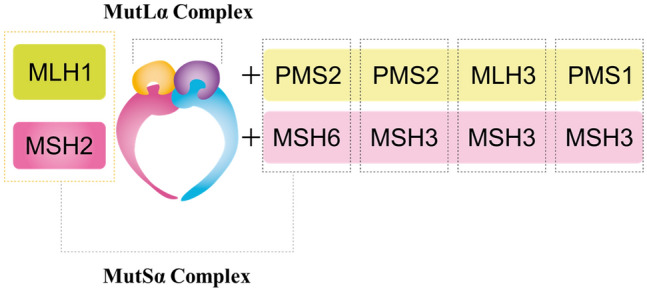
MMR protein human MutLα/MutSα complex. *MLH1* MutL homolog 1, *MSH2* MutS homolog 2, *PMS2* postmeiotic segregation increased 2, *MSH6* MutS homolog 6

The expression of MMR proteins (MLH1, MSH2, MSH6, and PMS2) in tumor tissue is examined by IHC to evaluate whether the tumor has dMMR. In the evaluation, an internal positive control (e.g., in colorectal cancer, the glandular base of the colonic mucosa or the germinal center of a lymphoid follicle in non-tumor tissue) is used to check the appropriateness of staining. If all four proteins are expressed, the tumor is determined to be pMMR, and if the expression of at least one protein is lost, the tumor is determined to be dMMR. An advantage of using IHC instead of MSI testing is that genes responsible for dMMR status can be presumed based on the pattern of proteins whose expression is lost. For example, MSH6 can form a heterodimer only with MSH2. Therefore, if the MSH2 gene is altered, MSH6 becomes unstable as the protein and becomes degraded, resulting in the loss of both MSH6 and MSH2 expressions in immunohistochemistry. In contrast, MSH2 can form a heterodimer with MSH3, as well as with MSH6. Therefore, even if the MSH6 gene is altered, MSH2 expression is maintained. Similarly, PMS2 can form a heterodimer only with MLH1, but MLH1 can form heterodimers with proteins other than PMS2 (Fig. 4). In many cases, the staining patterns in Table 6 are displayed. If a staining result does not show any of these patterns, check the appropriateness of staining. If a difficulty arises in judgment, perform additional testing such as MSI testing to make a comprehensive judgment.

**Table 6:** Suspected mutant genes in immunostaining for MMR proteins

	Expression in immunostaining			
	MLH1	MSH2	PMS2	MSH6
Mutant gene				
* MLH1*	–	+	–	+
* MSH2*	+	–	+	–
* PMS2*	+	+	–	+
* MSH6*	+	+	+	–

^*^When staining results other than the patterns in table are obtained, confirm the adequacy of staining before considering the possibility that the patient is exceptional and perform MSI testing if needed

It is recommended to evaluate four proteins, MLH1, MSH2, MSH6, and PMS2. However, if the evaluation of the four proteins is difficult because the amount of specimens is limited or for other reasons, screening only with MSH6 and PMS2 is acceptable [51].

In December 2021, a kit for detecting deficient mismatch-repair (MMR) function that consists of 4 IHC test kits, each of which detects an MMR protein expressed in tumor tissue (MLH1, MSH2, MSH6, and PMS2), was approved as an in vitro diagnostic in Japan.

#### NGS testing

The evaluation of deficient MMR function using the NGS techniques is broadly divided into methods that target only microsatellite regions and those that evaluate MMR function as a part of comprehensive cancer genome profiling. As an example of the former, the MSIplus panel has been reported [52]. This method measures the lengths of a total of 18 different microsatellite marker regions using the NGS technique. If instability is detected in 33% or more of the markers, the condition is judged to be MSI-H.

Examples of the latter are the FoundationOne^®^ CDx test and OncoGuide™ NCC oncopanel. The FoundationOne^®^ CDx test analyzes the length of repetitive sequences in approximately 2000 microsatellite regions, calculates an MSI score, and provides an assessment of MSI-high (MSI-H), MS-equivocal, or microsatellite-stable (MSS) based on criteria established in an equivalence study that compared it with polymerase chain reaction (PCR). If the assessment is MS-equivocal, an intermediate status between MSI-H and MSS, a confirmatory test is performed using an approved test such as another in vitro diagnostic [53]. The OncoGuide™ NCC oncopanel calculates an MSI score in microsatellites of up to five bases from mononucleotide repeats at 576 locations by comparing tumor tissue and blood cells (normal). An MSI score ≥ 30 is considered MSI-H (not approved as a companion diagnostic as of August 2021). Other methods include the MSIsensor algorithm using MSK-IMPACT [54], the MOSAIC algorithm using whole exome sequencing (WES) [12], and the MANTIS algorithm [55]. These methods determine a condition to be MSI-H differently depending on databases and algorithms regarding the regions to be profiled and the microsatellite markers located in the regions.

In June 2021, FoundationOne^®^ CDx was approved in Japan as a companion diagnostic for nivolumab and pembrolizumab in the treatment of cancers with high-frequency microsatellite instability (MSI-H).

### Immune checkpoint inhibitors for dMMR solid tumors

The PD-1 (CD279) molecule, which belongs to the CD28 family, is an immunosuppressive costimulatory signal receptor and was cloned by Honjo et al. in 1992 [56]. Subsequently, it was found that PD-1 is expressed in activated T cells and B cells and, in myeloid cells, inhibits T cell activity in an antigen-specific manner by binding to its ligand, and plays an important role in peripheral immune tolerance. PD-1 ligands include PD-L1 (CD274 and B7-H1) and PD-L2 (CD273 and B7-DC). The PD-1/PD-L1 pathway is the main immunoregulatory system utilized by cancer cells to escape T cell immunosurveillance and has been detected in various solid tumors. Another known immune checkpoint is cytotoxic T-lymphocyte-associated protein 4 (CTLA-4: CD152). Binding of CTLA-4 on cytotoxic T-cells in lymphatic tissue to CD80/86 on antigen-presenting cells inhibits T-cell activation.

Immune checkpoint-inhibiting monoclonal antibody drugs that are being introduced in clinical practice are anti-PD-1 antibody drugs (pembrolizumab and nivolumab), anti-PD-L1 antibody drugs (atezolizumab, avelumab, and durvalumab), and an anti-CTLA-4 antibody drug (ipilimumab). These drugs exert anti-tumor effects by reactivating anti-tumor immunity through the activation of tumor-specific cytotoxic T lymphocytes (CTL) in the tumor microenvironment. They exert anti-tumor effects through a mechanism of action different from those of conventional antineoplastic drugs.

In dMMR solid tumors, genomic alterations occur at a high frequency due to deficient MMR function. Such alterations lead to the synthesis of proteins with altered amino acids, parts of which are presented as antigenic peptides by the major histocompatibility complex (MHC). These new antigens, called neoantigens, are recognized as non-self and activate Th1/CTL in tumor tissues. On the other hand, the expression of immune checkpoint molecules including PD-1 is induced, as negative feedback.

Thus, the immune system plays an important role in the tumor control mechanism in dMMR solid tumors, and therefore, the efficacy of immune checkpoint inhibitors is expected.

The KEYNOTE-016 study was a phase II study to explore the efficacy and safety of pembrolizumab in patients with all solid tumors including colorectal cancer, and the outcomes from 86 patients with 12 types of dMMR solid tumors have been reported [10]. The outcomes were good with an objective response rate (ORR) of 53% (95% CI 42–64%) and a complete response (CR) of 21%. Neither median progression-free survival (PFS) nor median overall survival (OS) was reached, and no obvious differences were detected among different types of solid tumors [10].

Moreover, the KEYNOTE-164, a phase II study of pembrolizumab in patients with dMMR colorectal cancers, was conducted with two cohorts, i.e., patients who had previously received chemotherapy with fluoropyrimidines, oxaliplatin, and irinotecan (Cohort A) and those who had previously received one or more regimens of chemotherapy (Cohort B). The treatment outcomes of 61 patients in Cohort A were good with an ORR of 28% (95% CI 17–41), a median PFS of 2.3 months (95% CI 2.1–8.1), and the median OS not reached. The median duration of response (DoR) was not reached, and 82% of the patients who responded had a DoR of 6 months or longer [57]. Similarly, in the KEYNOTE-158 study, a phase II study of pembrolizumab in standard systemic treatment-unresponsive/intolerant patients with dMMR advanced solid tumors, the treatment outcomes of 94 patients were good with an ORR of 37% (95% CI 28–48), a median PFS of 5.4 months (95% CI 3.7–10.0), and a median OS of 13.4 months (95% CI 10.0–NR), demonstrating efficacy irrespective of cancer types. Moreover, the median DoR was not reached, and 51% of the patients who responded had a DoR of 6 months or longer, demonstrating the sustained efficacy [58]. Regarding adverse events, unlike conventional anticancer drugs, not only adverse events such as arthritis, nausea, malaise and pruritus but also unique autoimmune disease-like immune-related adverse events (irAEs) may occur. Therefore, careful management is required.

### Lynch syndrome

Lynch syndrome is an autosomal dominant hereditary disease caused by pathogenic variants of the MMR gene in the germline. According to reports from Europe and the USA, it accounted for 2–4% of all colorectal cancer and is associated with a variety of malignancies in patients and families, particularly colorectal cancer and endometrial cancer (Table 7). However, because it also enables variety of cancer prevention measures to be taken, its diagnosis is clinically important.

**Table 7:** Cumulative lifetime risk of lynch syndrome-associated neoplasms

Cancer type	Cumulative risk (%)
Colorectal cancer	54–74 (male), 30–52(female)
Endometrial cancer	28–60
Gastric cancer	5.8–13
Ovarian cancer	6.1–13.5
Small-bowel cancer	2.5–4.3
Bile duct cancer	1.4–2.0
Pancreatic cancer	0.4–3.7
Urothelial cancer	3.2–8.4
Brain tumor	2.1–3.7
Sebaceous gland tumor	1–9*

^*^Partial Amendment of JSCCR Guidelines 2016 for the Clinical Practice of Hereditary Colorectal Cancer

In patients with Lynch syndrome, one allele of the MMR gene has a pathogenic variant of the germline. If the other wild-type allele acquires a loss-of-function alteration (including methylation in the promoter region), MMR function is lost, and this is considered to contribute to canceration [1].

In Japan, if clinical information of a patient meets the Amsterdam Criteria II or the revised Bethesda Guidelines, MSI testing or IHC is recommended for the secondary screening (Supplementary Fig. 1) [59]. In Europe and the USA, a universal screening in which MSI testing or IHC is performed in all (or ≤ 70-year-old) patients with colorectal cancer or endometrial cancer, irrespective of the presence of findings suggesting that Lynch syndrome has been proposed [60, 61].

If the result of MSI testing or IHC suggests Lynch syndrome, the genetic testing of the MMR gene should be considered for definitive diagnosis. If genetic testing is conducted, it is recommended to properly select subjects to be tested (the patient and relatives) and to provide them with genetic counseling before and after genetic testing. Some patients have genetic alterations that are not detectable by the current genetic testing methods, and a definitive diagnosis of Lynch syndrome cannot be made in these patients. Therefore, results should be interpreted carefully. If Lynch syndrome is diagnosed, effort is made to prevent cancer in blood relatives and others through genetic counseling.

[Note] Usefulness of *BRAF* testing in patients who were determined to be dMMR by dMMR testing.

The main reason for sporadic colorectal cancers to become dMMR is an acquired abnormal methylation in the promoter region of the *MLH1* gene. In these cancers, the loss of MLH1/PMS2 protein expression is detected by immunohistochemistry. In 35–43% of MSI-H colorectal cancers, the *BRAF* V600E mutation is detected [18], while in colorectal cancers in patients with Lynch syndrome, almost no *BRAF* V600E mutations are detected even in MSI-H cancers [12]. Therefore, in the medical care for colorectal cancer, if the dMMR testing result shows MSI-H or the loss of MLH1/PMS2 expression, checking for the *BRAF* V600E mutation helps distinguish Lynch syndrome-related colorectal cancers from sporadic ones [62]. However, caution is needed because it has been reported that the *BRAF* V600E mutation was detected in some colorectal cancers that developed in patients with Lynch syndrome attributable to the *PMS2* gene. For solid tumors other than colorectal cancer, the usefulness of a differential diagnosis with *BRAF* V600E mutation has not been reported.

[Note] Constitutional mismatch repair deficiency (CMMRD).

CMMRD syndrome, in which pathogenic variants are seen in both alleles of an MMR gene (homozygous or compound heterozygous), results in a predisposition to childhood cancer. It is mainly associated with hematopoietic, central nervous system, and colorectal malignancies. It is often associated with skin findings that resemble those of neurofibromatosis type 1 (NF1) and therefore must be differentiated from that condition [63]. Since Turcot et al. described siblings with familial colorectal polyposis in association with brain tumors in 1959, the condition in which colorectal tumor and brain tumor are concurrently seen has been referred to as Turcot syndrome, and it is likely that some cases of CMMRD have been diagnosed as Turcot syndrome. CMMRD was first verified by molecular genetic methods in 1999, with many hypermutants with MSI-H seen in the tumors of patients with CMMRD and an overwhelmingly large number of neoantigens expressed in such tumors. Recently, anti-PD-1/PD-L1 antibody drugs have been reported to be effective in CMMRD [64, 65].

### Clinical questions (CQs)

The following requirements have been prepared regarding the dMMR testing performed to select patients who are likely to benefit from PD-1/PD-L1 inhibitors and the administration of them. The clinical recommendations propose the following eight requirements in two CQs regarding the dMMR testing performed to select patients who are likely to benefit from anti-PD-1/PD-L1 antibody drugs.

1. For patients with unresectable advanced or recurrent solid tumors other than those for which immune checkpoint inhibitors can be used clinically irrespective of MMR function, dMMR testing is strongly recommended to determine whether immune checkpoint inhibitors are indicated.
2. For patients with unresectable solid tumors for which immune checkpoint inhibitors can already be used clinically irrespective of MMR function, dMMR testing should be considered to determine whether immune checkpoint inhibitors are indicated.
3. For patients with solid tumors that are curable with local treatment, dMMR testing is not recommended to determine whether immune checkpoint inhibitors are indicated.
4. For patients with unresectable solid tumors for which an immune checkpoint inhibitor has already been used, dMMR testing is not recommended to determine again whether immune checkpoint inhibitors are indicated.
5. For a tumor occurring in patients who have already been diagnosed with Lynch syndrome, dMMR testing is strongly recommended to determine whether immune checkpoint inhibitors are indicated.
6. As dMMR testing to determine whether immune checkpoint inhibitors are indicated, microsatellite instability (MSI) testing is strongly recommended.
7. As dMMR testing to determine whether immune checkpoint inhibitors are indicated, immunohistochemistry (IHC) testing is strongly recommended.
8. As microsatellite instability (MSI) testing to determine whether immune checkpoint inhibitors are indicated, a next-generation sequencing (NGS) test whose analytical validity has been established (by receiving regulatory approval, etc.) is strongly recommended.

Please keep in mind that these clinical recommendations will be revised in a timely manner, along with continuously and steadily advancing cancer treatment and new knowledge on biomarkers.

We will explain each CQ in detail.

#### CQ1: Patients for whom dMMR testing is recommended

PubMed was searched using the following queries: “MSI or microsatellite instability or MMR or mismatch repair,” “neoplasm,” and “tested or diagnos* or detect*.”The same queries were used to search Cochrane Library. For the search period from January 1980 to January 2021, 985 articles were extracted from PubMed and 57 from Cochrane Library. In addition, two articles were retrieved by handsearching. In the primary screening, 380 articles were extracted, and 347 were extracted in the secondary screening. A qualitative systematic review of these articles was then performed.
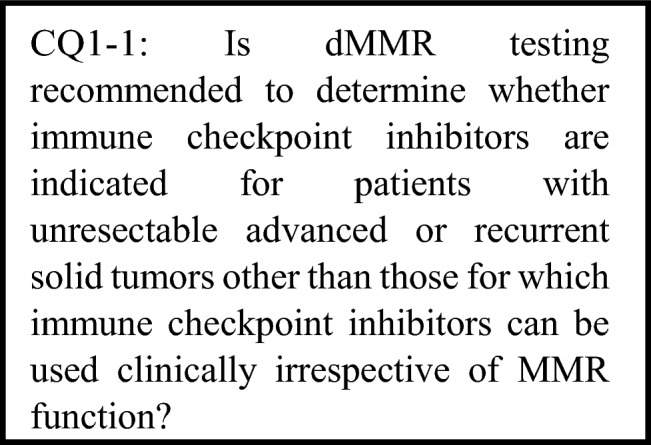


For patients with unresectable advanced or recurrent solid tumors other than those for which immune checkpoint inhibitors can be used clinically irrespective of MMR function, dMMR testing is strongly recommended to determine whether immune checkpoint inhibitors are indicated.

Recommendation level: Strongly recommended [SR: 19, R: 1, ECO: 0, NR: 0].

Based on the results of a pooled analysis of 149 patients with advanced/recurrent dMMR solid tumors that progressed after chemotherapy from five clinical studies of pembrolizumab (KEYNOTE-016 study, KEYNOTE-164 study (Cohort A), KEYNOTE-012 study, KEYNOTE-028 study, and KEYNOTE-158 study), the United States Food and Drug Administration (FDA) approved pembrolizumab for dMMR solid tumors including colorectal cancers that are resistant to standard systemic treatment or for which no standard treatment is available, on May 23, 2017. In Japan, pembrolizumab was approved on December 21, 2018, based on the updated results of the KEYNOTE-164 study (Cohort A) and KEYNOTE-158 study (Table 8).

**Table 8:** Results of the KEYNOTE-164 study (Cohort A) and KEYNOTE-158 study

	*N*	Response rate *n* (%)
Colorectal cancer	61	17 (28)*
Non-colorectal cancer	94	35 (37)**
Endometrial cancer	24	13 (54)
Gastric cancer	13	6 (46)
Small-bowel cancer	13	4 (31)
Pancreatic cancer	10	1 (10)
Bile duct cancer	9	2 (22)
Adrenocortical cancer	3	1 (33)
Mesothelioma	3	0 (0)
Small cell lung cancer	3	2 (67)
Cervical cancer	2	1 (50)
Neuroendocrine carcinoma	2	0 (0)
Thyroid cancer	2	0 (0)
Urothelial cancer	2	1 (50)
Brain tumor	1	0 (0)
Ovarian cancer	1	0 (0)
Prostate cancer	1	0 (0)
Retroperitoneal tumor	1	1 (100)
Salivary gland cancer	1	1 (100)
Sarcoma	1	1 (100)
Testicular tumor	1	0 (0)
Tonsil cancer	1	1 (100)

^*^ORR for dMMR colorectal cancer 95%CI 17–41%

**ORR for dMMR non-colorectal cancer 95%CI 28–48%

A study of nivolumab monotherapy and nivolumab + ipilimumab (an anti-CTLA-4 antibody drug) combination therapy in patients with dMMR colorectal cancers (CheckMate-142 study) reported good outcomes with the ORRs of 31% and 55%, respectively, and the median PFSs was not reached in either group [66, 67]. A therapeutic effect was observed irrespective of the degree of PD-L1 expression, the presence of the *BRAF*/*KRAS* mutations, or the presence of Lynch syndrome. Patient evaluation using EORTC QLQ-C30 demonstrated improved QOL and clinical symptoms [66, 67]. Based on these results, the FDA approved nivolumab monotherapy in August 2017 and nivolumab + ipilimumab combination therapy in July 2018 for metastatic dMMR colorectal cancers that progressed after chemotherapy including fluoropyrimidine antineoplastic agents. Also in Japan, based on the results of the same study, nivolumab monotherapy was approved in February 2020 and nivolumab + ipilimumab combination therapy was approved in September 2020 for the same population. For durvalumab, an anti-PD-L1 antibody drug, a phase II study in patients with dMMR colorectal cancers and phase I/II studies in patients with dMMR solid tumors were conducted and demonstrated an efficacy with the ORR for colorectal cancers of 22% and an overall ORR of 23% [68]. Efficacy for dMMR solid tumors was reproduced in case reports and the analyses of dMMR subgroups in prospective phase II studies.

As for dMMR colorectal cancer, the KEYNOTE-164 study reported good outcomes not only in patients who had received chemotherapy with fluoropyrimidines, oxaliplatin, and irinotecan hydrochloride hydrate (Cohort A), but also in 63 patients who had received one or more regimens of chemotherapy (Cohort B) with the ORR of 32% (95% CI 21–45), the median PFS of 4.1 months (95% CI 2.1–NR), and the median OS not reached. In addition, the phase III KEYNOTE-177 study was conducted to examine the efficacy of standard therapy and pembrolizumab monotherapy in untreated and unresectable advanced or recurrent colorectal cancer. Median PFS, the primary endpoint, was 16.5 months (95% CI 5.4–32.4) in the pembrolizumab group and 8.2 months (95% CI 6.1–10.2) in the standard therapy group, PFS being significantly longer in the pembrolizumab group (HR 0.60; 95% CI 0.45–0.80; *P* = 0.0002). The ORR was higher in the pembrolizumab group than in the standard therapy group: 43.8% (95% CI 35.8–52.0) and 33.1% (95% CI 25.8–41.1), respectively [69]. Median OS was not reached in the pembrolizumab monotherapy group (95% CI 49.2–NR) and was 36.7 months (95% CI 27.6–NR) in the standard therapy group (HR 0.74; 95% CI 0.53–1.03; *P* = 0.0359) [70]. Although a favorable trend was seen in the pembrolizumab monotherapy group, the difference was not significant. One reason for this may have been that 60% of the patients in the standard therapy group were administered an immune checkpoint inhibitor as a subsequent treatment. Based on the results of this study, pembrolizumab was approved by the FDA in June 2020 as the first-line therapy for unresectable advanced or recurrent dMMR colorectal cancer. In Japan, its indications were expanded on August 25, 2021, to include unresectable advanced or recurrent colorectal cancer with high-frequency microsatellite instability (MSI-H).

The efficacy of nivolumab–ipilimumab combination therapy in untreated dMMR colorectal cancer also was examined in the CheckMate-142 study. The ORR in that study was 60% (95% CI: 44.3–74.3), indicating a favorable anti-tumor effect [71]. In addition, phase III studies comparing standard therapy and PD-1/PD-L1 antibody drugs in untreated dMMR colorectal cancer are being conducted (COMMIT study, CheckMate-8HW study), and the results are expected.

The molecular biology evidence also suggests that a high level of immunogenicity is common to dMMR solid tumors, and although the studies have lacked adequate sample sizes for each type of cancer and treatment line, they are showing immune checkpoint inhibitors to be effective in such cancers. However, it should be noted that the effect of immune checkpoint inhibitors is not consistently seen in some types of dMMR solid tumors (e.g., gliomas) [72].

As for adverse events, although caution is required for serious immune-related adverse events, which occur frequently, they are generally tolerable. Therefore, for all patients with dMMR solid tumors, including tumors for which immune checkpoint inhibitors have no approved organ-specific indications from the viewpoint of efficacy and safety, immune checkpoint inhibitors can be a potent treatment option. When cancer worsens, the patient’s general condition also often worsens. Consequently, in view of the turnaround time (TAT) for dMMR testing, it is advisable to perform such testing and determine whether the patient has an indication for an immune checkpoint inhibitor early in the process of diagnosis. In colorectal cancer, an assessment is needed before treatment begins. Depending on the type of cancer, certain biomarker tests are needed in order to decide on a treatment strategy (e.g., *RAS*/*BRAF* testing for colorectal cancer; an HER2 testing for gastric cancer; and EGFR, ALK, ROS1, and PD-L1 expression testing for non-small-cell lung cancer). Although simultaneous testing is desirable, the relative priority of the biomarkers also must be considered.

Based on the above considerations, dMMR testing is strongly recommended to determine whether the use of an immune checkpoint inhibitor is indicated for patients with unresectable advanced or recurrent solid tumors.
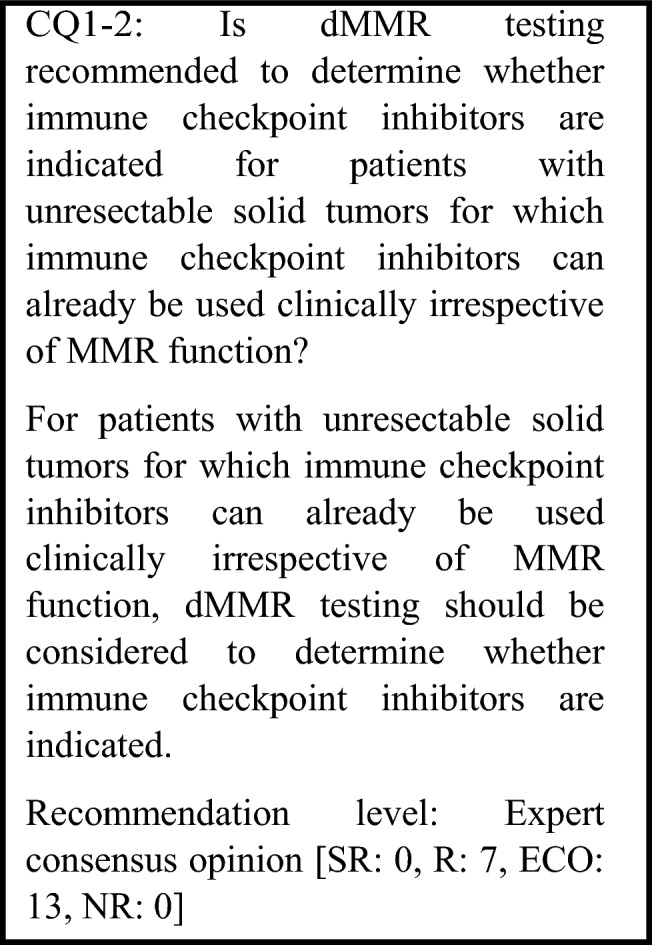


Irrespective of MMR function, dMMR testing is generally considered unnecessary in solid tumors for which an immune checkpoint inhibitor can be used irrespective of MMR function, because determining whether such use is indicated does not depend on MMR function. However, when patients with a solid tumor for which whether an immune checkpoint inhibitor is indicated is determined by non-dMMR biomarkers, such as PD-L1 expression, test negative for these biomarkers, the effectiveness of immune checkpoint inhibitors can be expected if dMMR is present. Therefore, dMMR testing is recommended.
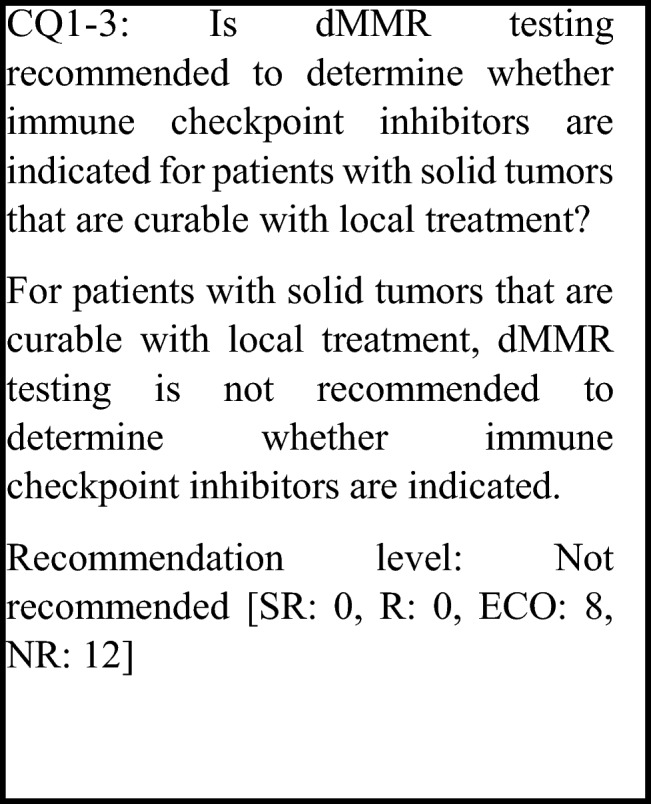


For malignant melanoma, an anti-PD-1 antibody drug has demonstrated efficacy as postoperative adjuvant therapy and has been approved (KEYNOTE-054 study [73] and ONO-4538-21 study [74]). For non-small cell lung cancer, an anti-PD-L1 antibody drug has received regulatory approval based on the results of the PACIFIC study, a randomized, double-blind, placebo-controlled, multicenter phase III study of the anti-PD-L1 antibody drug administered sequentially in patients with unresectable locally advanced cancer (stage III) who did not show disease progression after curative concurrent chemoradiotherapy (CRT) using platinum drugs [75]. In the Checkmate-577 study, the efficacy of nivolumab as postoperative adjuvant therapy was shown in stage II/III esophageal and gastroesophageal junction cancer that was resected after preoperative chemoradiotherapy [76]. However, since no difference in efficacy due to MMR function has been reported from these studies, dMMR testing before treatment is not necessary in principle. For other solid tumors, the efficacy of immune checkpoint inhibitors as perioperative treatment has not been established. Therefore, if the tumor is curable with local therapy, dMMR testing to select therapeutic drugs is not necessary in principle. Thus, at present, for patients with solid tumors that are not locally advanced or metastatic, dMMR testing for determining whether immune checkpoint inhibitors are indicated is not recommended.

However, dMMR is a favorable prognostic factor for colorectal cancer, particularly for stage II colorectal cancer, and if dMMR is present, adjuvant chemotherapy with fluoropyrimidines is unnecessary [77, 78]. Therefore, it is considered to be desirable to perform dMMR testing to determine whether to administer adjuvant chemotherapy (for details, refer to the “Japanese Society of Medical Oncology Clinical Guidelines: Molecular Testing for Colorectal Cancer Treatment, 4th edition”) [79].

Furthermore, a study examining the efficacy of combination therapy with FOLFOX and atezolizumab as postoperative adjuvant chemotherapy for Stage III dMMR colorectal cancer (ATOMIC, Alliance A021502) is currently being conducted. In addition, many studies examining the efficacy of perioperative treatment with immune checkpoint inhibitors and the use of combined chemoradiotherapy in locally advanced cancers are currently being conducted. If good outcomes are obtained from these studies, dMMR testing will be necessary for solid tumors curable with local treatment. The need for such testing will be examined for each type of cancer in multidisciplinary team (MDT) conferences.
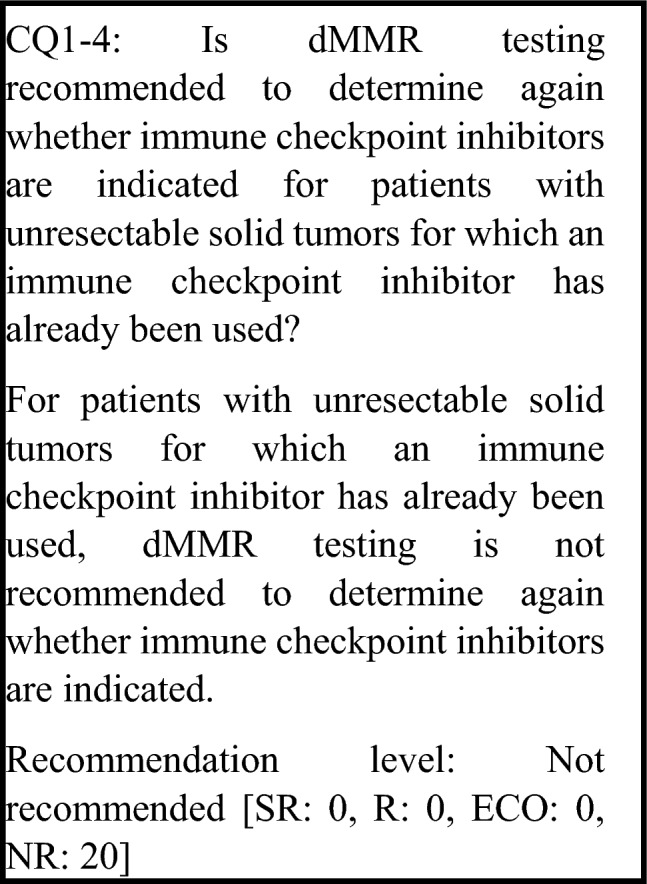


Immune checkpoint inhibitors have been approved for use in some solid tumors, irrespective of MMR function. There has been some reporting on the effectiveness of administering a different immune checkpoint inhibitor when an immune checkpoint inhibitor has already been administered. A study that retrospectively examined patients with non-small-cell lung cancer who received nivolumab as the first-line therapy and an anti-PD-1 antibody as the second-line therapy and beyond, found that efficacy was significantly greater in patients who received nivolumab, the first-line therapy, for 3 months or longer [80]. However, this has not been examined in a prospective study, and differences in efficacy according to MMR function have not been shown. Therefore, dMMR testing is not recommended for the purpose of administering an immune checkpoint inhibitor in patients with solid tumors for which an immune checkpoint inhibitor has already been used.
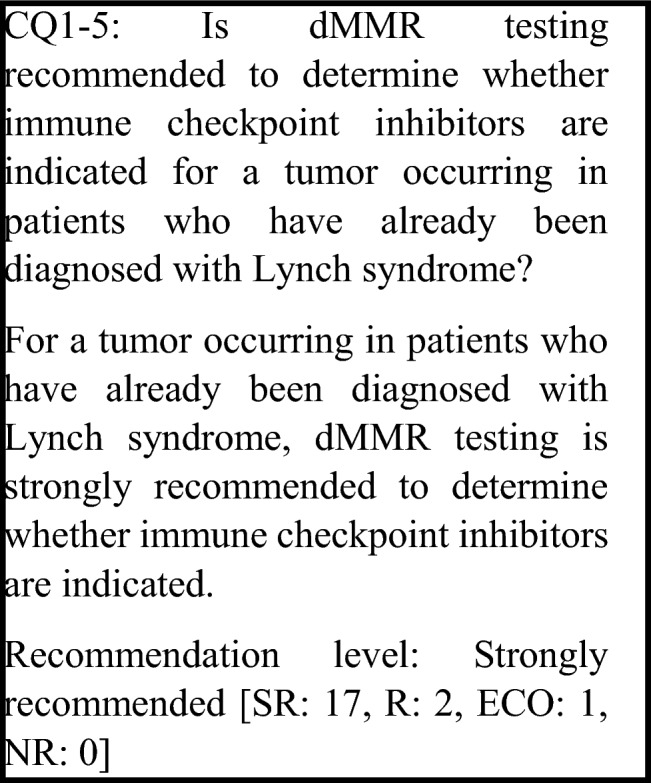


Although the incidence of dMMR is high (80–90%) [81] in colorectal cancer that occurs in patients with Lynch syndrome, pMMR tumors are also seen, although rarely, among the tumors that occur in such patients. There is currently no clear evidence regarding the sensitivity of immune checkpoint inhibitors when pMMR is present in the tissues of patients with Lynch syndrome. In view of this, dMMR testing is strongly recommended to determine whether immune checkpoint inhibitors are indicated for a tumor occurring in patients with Lynch syndrome.

#### CQ2: Testing methods of dMMR

PubMed was searched using the following queries: “MSI or microsatellite instability or MMR or mismatch repair,” “neoplasm,” “IHC or immunohistochemistry,” “PCR or polymerase chain reaction,” and “NGS or next generation sequencer.” The same queries were used to search Cochrane Library. For the search period from January 1980 to January 2021, 1031 articles were extracted from PubMed and 120 from Cochrane Library. In the primary screening, 669 articles were extracted, and 537 were extracted in the secondary screening. A qualitative systematic review of these articles was then performed.
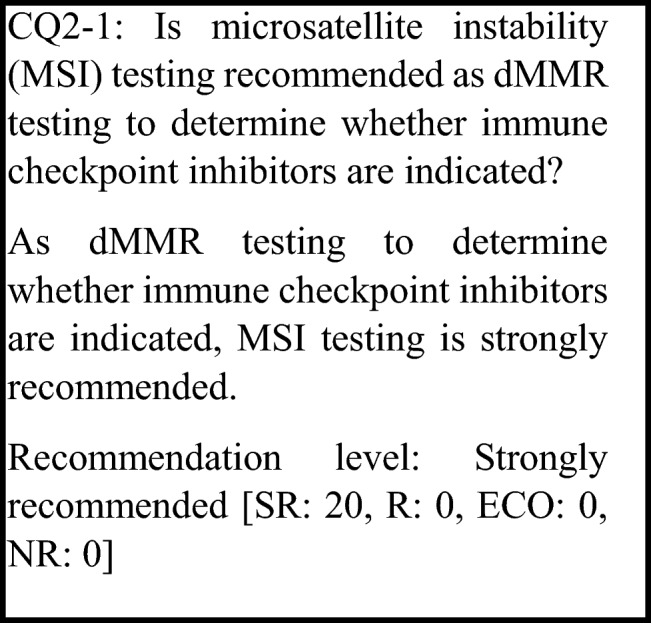


The pooled analysis of patients with dMMR from five KEYNOTE studies [KEYNOTE-016 study, KEYNOTE-164 study (Cohort A), KEYNOTE-012 study, KEYNOTE-028 study, and KEYNOTE-158 study] that enrolled patients who were determined to be dMMR based on IHC or MSI testing performed at each study site demonstrated good anti-tumor effect of pembrolizumab. Among 149 patients, 60 patients were determined to be dMMR by MSI testing alone, 47 patients by IHC alone, and 42 patients by both tests [82]. Among them, only 14 patients were determined to be MSI-H by MSI testing performed at a central testing laboratory. A phase II study of nivolumab in patients with colorectal cancer who were determined to be dMMR (CheckMate-142 study) enrolled patients who were determined to be dMMR by IHC or MSI testing performed at each study site and has demonstrated the efficacy of nivolumab + ipilimumab [66]. Thus, if a cancer is determined to be dMMR by either IHC or MSI testing, the anti-tumor effect of immune checkpoint inhibitors is expected, although there may be some differences depending on the type of cancer.

In Japan, in September 2018, “MSI test kit (FALCO)” received regulatory approval as a companion diagnostic for pembrolizumab. As of June 2021, it had been approved for the following indications: “assistance in evaluating whether pembrolizumab is indicated in patients with solid tumors,” “assistance in evaluating whether nivolumab is indicated in patients with cancer of the colon or rectum,” “assistance in diagnosing Lynch syndrome in colorectal cancer,” and “assistance in selecting chemotherapy for colorectal cancer.” Any institution in Japan can order this test, and the test is performed in quality-assured testing facilities. Moreover, this test kit can determine the dMMR status by testing tumor tissue alone, which is therefore very convenient [48]. Thus, as a dMMR testing method for determining whether immune checkpoint inhibitors are indicated, MSI testing is strongly recommended.
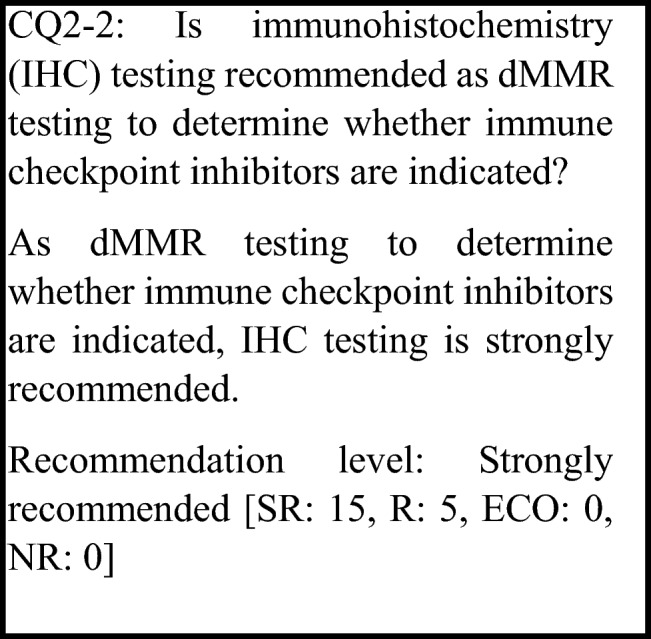


As mentioned above, the efficacy of immune checkpoint inhibitors was demonstrated in patients enrolled in the pooled analysis of five KEYNOTE studies and those in the Checkmate-142 study, who were diagnosed as having dMMR based on IHC or MSI testing performed at each study site. In both analyses, the efficacy of immune checkpoint inhibitors was demonstrated also in patients who were determined to be dMMR by IHC testing alone. Actually, in the Checkmate-142 study, in which MSI was determined centrally by MSI testing (with 5 markers used in the Bethesda panel and TGF-beta receptor type 2), 14 of the 74 patients who were determined to be dMMR by IHC testing at each study site were judged to be non-MSI-H. However, 3 of the 14 patients (21%) responded to treatment [66], and this fact suggests that even when the results of the IHC testing and MSI testing are not consistent and the dMMR was diagnosed based only on one test, the anti-tumor effect of immune checkpoint inhibitors can be expected. Compared to MSI testing and NGS testing, IHC can be performed inexpensively at individual medical institutions. In December 2021, a kit for detecting deficient mismatch-repair (MMR) function that consists of 4 IHC test kits, each of which detects an MMR protein expressed in tumor tissue (MLH1, MSH2, MSH6, and PMS2), was approved as an in vitro diagnostic in Japan. Based on the above considerations, IHC testing is strongly recommended as dMMR testing to determine whether immune checkpoint inhibitors are indicated.

While a high concordance rate between MSI testing results and IHC testing results has been reported [83, 84], some inconsistent cases have been reported. One example is pathogenic missense variants of the MMR genes [85, 86]. In this case, proteins that have lost MMR function are expressed. Therefore, the MSI testing result indicates MSI-H, and the tumor is determined to be dMMR, while in IHC testing, MMR proteins are detected, and the tumor is determined to be pMMR (false negative). For this dMMR tumor, immune checkpoint inhibitors are presumed to be effective. It has been reported that such missense variants are observed in approximately 5% of patients with Lynch syndrome [87]. Since possible causes of false-negative cases by MSI testing include a low tumor cell ratio, a tumor cell ratio of ≥ 50% is recommended for the MSI test (FALCO). On the other hand, the positive predictive value of IHC or MSI testing has been reported to be ≥ 90% [84]. It has been reported that when patients who were diagnosed with dMMR solid tumors by IHC or MSI testing and received immune checkpoint inhibitors but did not respond to the therapy were evaluated again by both MSI testing and IHC testing, 60% of them were found to be MSI-L/MSS/pMMR [80]. In IHC testing, there are some staining patterns, such as a case of partially reduced protein expression, for which assessment methods have not yet been specified. In addition, it needs to be considered which is more recommended between MSI testing and IHC testing according to the condition of the specimen. In order to extensively identify patients who can benefit from immune checkpoint inhibitors, testing should be performed based on a good understanding of the characteristics of both tests. If a false-positive or false-negative result is expected or if there are doubts about the precision or results of the test, performing the other test should be considered.
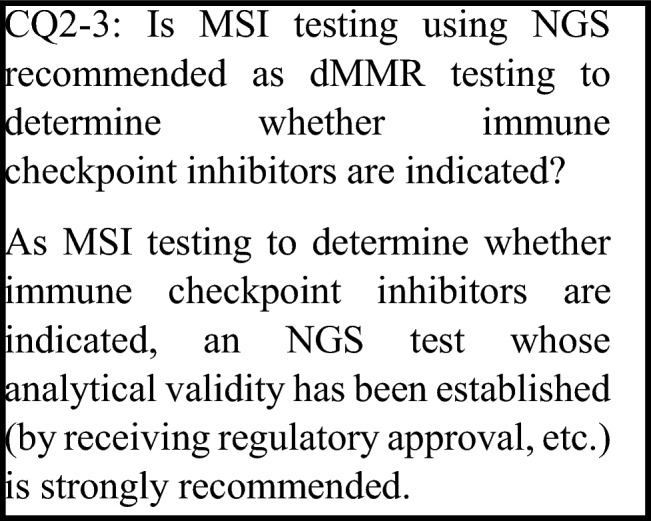


Recommendation level: Strongly recommended [SR: 14, R: 6, ECO: 0, NR: 0].

In Japan, on December 27, 2018, the FoundationOne^®^ CDx received marketing approval for obtaining comprehensive cancer genome profiles of a tumor tissue from patients with solid tumors and for detecting somatic cell genetic alterations to determine the indication of some molecular targeted drugs. Because FoundationOne^®^ CDx includes MSI testing using the NGS method, the comprehensive cancer genome profiling and MSI testing (the NGS method) can be performed simultaneously for each cancer type with specimens and at the timing specified in the latest guidelines and other documents issued by relevant academic societies. In June 2021, FoundationOne^®^ CDx was approved in Japan as a companion diagnostic for nivolumab and pembrolizumab in the treatment of cancers with a high-frequency microsatellite instability (MSI-H). Also in June 2021, the OncoGuide™ NCC oncopanel system was upgraded (v2.01), enabling MSI assessment. However, as of August 2021, no tests other than FoundationOne^®^ CDx were actually being used as companion diagnostics. In its administrative document titled Points to Consider Regarding National Health Insurance Coverage of Gene Panel Tests, the Ministry of Health, Labour and Welfare states that if, after a gene panel test is performed, an expert panel determines that “administration of a drug related to genetic alterations for which a companion test exists is appropriate” based on references such as the package insert, guidelines, or the scientific literature, that drug can be administered without repeating the companion test. Because there are requirements for facilities to perform these NGS tests, microsatellite instability assessment using the NGS method can be accessed at limited facilities in Japan. In addition, NGS testing has a certain failure rate, which poses a feasibility issue for such testing.

In the five KEYNOTE studies and the Checkmate-142 study conducted for the application for the FDA approval of pembrolizumab, screening tests for dMMR did not include NGS testing. However, the determination of MMR function using NGS testing and MSI testing has a similar measurement principle in that a repeat number of microsatellites is used to determine whether a tumor has dMMR, and it has been reported that the concordance rates between these tests were extremely high, 99.4% in colorectal cancers and 96.5% in solid tumors other than colorectal cancers [88]. Moreover, when inconsistent cases were analyzed, they were dMMR by IHC testing but MSS by NGS testing, suggesting that NGS testing is more useful. Therefore, it is scientifically unnecessary to perform testing using the MSI test kit (FALCO), a companion diagnostic, or IHC testing to reconfirm the status determined to be MSI-H by NGS testing, for which analytical validity has been established in the determination of MSI. Thus, as MSI testing to determine whether the use of immune checkpoint inhibitors is indicated, an NGS test whose analytical validity has been established (by receiving regulatory approval, etc.) is strongly recommended.

## Conclusion

Many clinical trials have reported the efficacy of immune checkpoint inhibitors in the treatment of dMMR advanced solid tumors. In this guideline, the panel recommends the requirements for performing dMMR testing properly to select patients who are likely to benefit from immune checkpoint inhibitors.

### Supplementary Information

Below is the link to the electronic supplementary material.

Supplementary file1 (DOCX 123 KB)
