# Supplementary figures and images for: Japanese Society of Medical Oncology/Japan Society of Clinical Oncology/Japanese Society of Pediatric Hematology/Oncology-led clinical recommendations on the diagnosis and use of immunotherapy in patients with DNA mismatch repair deficient (dMMR) tumors, third edition
Source: Int J Clin Oncol. 2023 Aug 20;28(10):1237–58. doi: 10.1007/s10147-023-02397-9 (PMC10542286; doi:10.1007/s10147-023-02397-9)

**Supplemental Figure 1. Diagnostic process for Lynch syndrome**


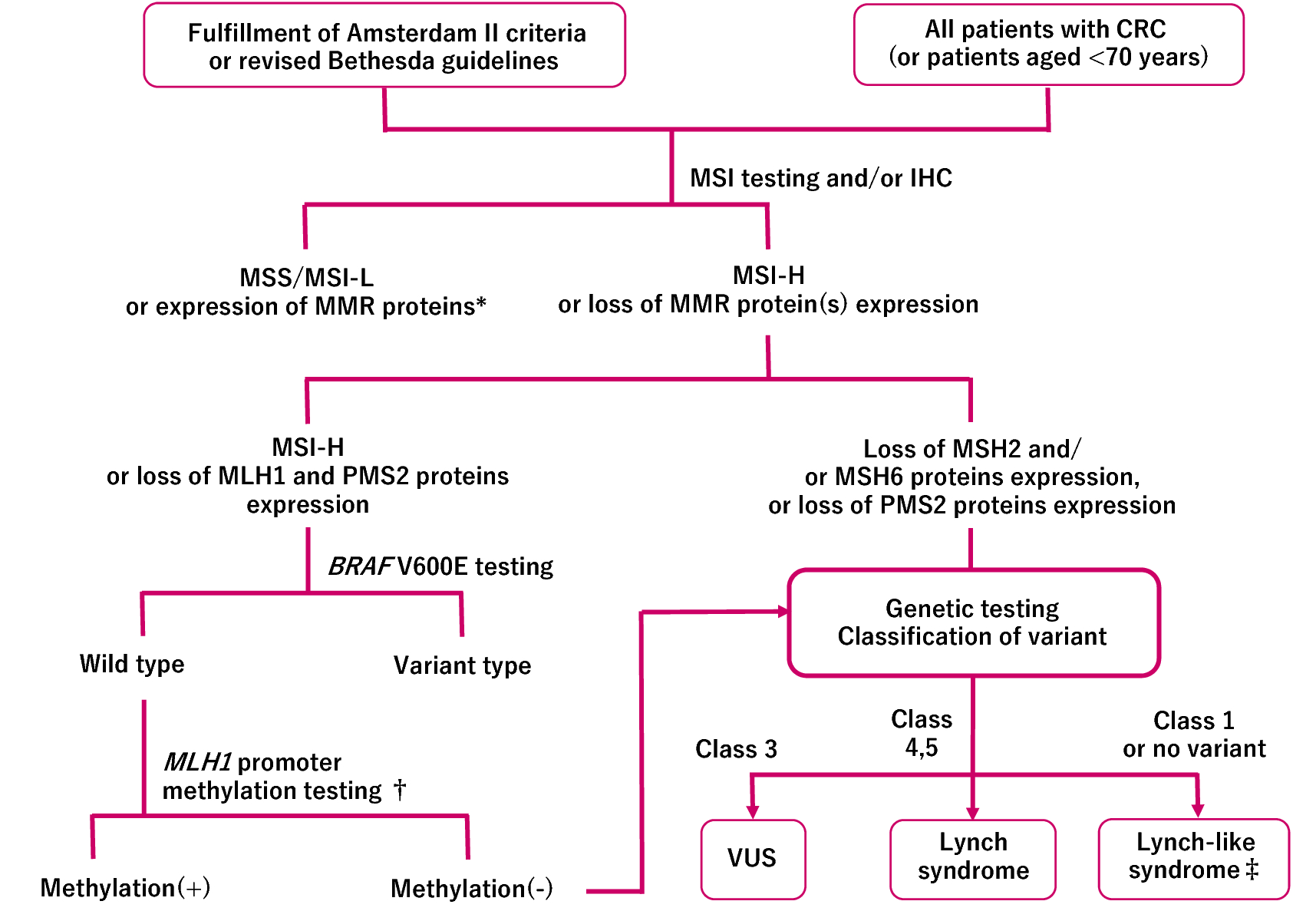

Supplement: Supplementary file 1 — Supplementary file1 (DOCX 123 KB) [file 10147_2023_2397_MOESM1_ESM.docx]
